# Dynamic expression and functional analysis of circRNA in granulosa cells during follicular development in chicken

**DOI:** 10.1186/s12864-019-5462-2

**Published:** 2019-01-30

**Authors:** Manman Shen, Tingting Li, Genxi Zhang, Pengfei Wu, Fuxiang Chen, Qiuhong Lou, Lan Chen, Xuemei Yin, Tao Zhang, Jinyu Wang

**Affiliations:** 1 College of Animal Science and Technology, Yangzhou University, Yangzhou, 225009 China; 2 Jiangsu Institute of Poultry Science, Chinese Academy of Agricultural Science, Yangzhou, 225216 China

**Keywords:** Circular RNA, Granulosa cells, Follicles, Chicken, Pathways

## Abstract

**Background:**

Circular RNA (circRNA) is a type of noncoding RNA involved in a variety of biological processes, especially in post-transcriptional regulation. The granulosa cells of follicles play a determining role in ovarian development. However, the function of circRNA in chicken follicles is unclear. To better understand the molecular mechanism underlying follicular development and granulosa cell function, we performed a strategy of second-generation sequencing and linear RNA depletion for granulosa cells from small yellow follicles (SYF, 5–8 mm), the smallest hierarchal follicles (F6, 9–12 mm), and the largest hierarchal follicles (F1, ~ 40 mm).

**Results:**

We predicted a total of 11,642 circRNAs that distributed on almost all chromosomes. The majority of the splice lengths of circRNAs were 200–500 nt and mainly produced from intron and CDS regions. During follicle growth, differentially expressed (DE) circRNAs showed dynamic changes which were tissue- and stage-specific. The host genes of DE circRNAs were functionally enriched in GTPase activity and several pathways involved in reproduction. Moreover, bioinformatic prediction analysis for *circRalGPS2* demonstrated that circRNAs from the same genes may share common miRNA to act as a sponge. The predicted target genes were enriched in various biological processes including cognition, cell communication, and regulation of signaling, and several pathways related to reproduction such as tight junction, oocyte meiosis, progesterone-mediated oocyte maturation, and *GnRH* signaling.

**Conclusions:**

This study provides a starting point for further experimental investigations into chicken circRNAs and casts a light on the understanding of follicle development.

**Electronic supplementary material:**

The online version of this article (10.1186/s12864-019-5462-2) contains supplementary material, which is available to authorized users.

## Background

Follicle development is a key determinant of reproduction performance and is affected by physiology and the environment. Despite a different follicle situation between birds and mammals, the characteristics of maturing hen ovarian follicles are equivalent to those occurring in mammals, thus hen ovarian follicles can be used as a model for the study of follicle development [1]. Ovulation is a process that occurs via the initial recruitment of primordial follicles, which is followed by primary follicles, small white follicles, larger white follicles, small yellow follicles (SYFs), and hierarchal follicles that progress toward maturity [2]. One of the striking characteristics of the bird follicle is the presence of 5–7 hierarchal follicles before ovulation. Hierarchal follicles develop from a pool of SYFs each day during the peak laying period [3].

The basic structure of the follicular wall consists of the theca and granulosa. The ovaries can increase in follicle number when treated by exogenous follicle-stimulating hormone (FSH) [4–6]; considering that FSH receptor (FSHR) mRNA in the theca layer changes little during follicle development [6], it is concluded that the granulosa cell (GC) contributes as a decisive factor in follicle development. The development of GCs involves multiple complex biological processes, affected by many molecular components including those at the genome and RNA transcript level [7, 8]. The expression of AMH mRNA increases with follicle development, and markedly decreases during follicle selection [2] and may affect SYF number [9]. It has been shown that epidermal growth factor (EGF) suppresses GC differentiation and decreases the abundance of FSHR mRNA [10]. FOXL2 plays different roles in prehierarchical GCs and pre-ovulatory GCs [11]. In brief, the growth, differentiation, and function of GCs are dependent on FSH and related functional genes. Elucidating the features of the molecular mechanism during GC development could provide a reference to understanding molecular development and improving assisted reproduction in humans.

There are two types of cellular RNA during transcription processes: coding and noncoding. In recent years, more and more noncoding RNA investigations have attracted researchers focused in this area. Notably, circular RNA (circRNA) is becoming a hot topic in life science since Salzman identified ~ 80 circRNAs in 2012 [12]. The circRNA exhibit characteristics without a 5′-cap structure or 3′-polyadenylated tail, mainly derived from exons, introns, intergenic, and antisense transcripts and are widespread in mammals and plants [13, 14]. The circRNA are recognized as having the following biological functions: an miRNA sponge, transcription template, regulating gene transcription, and RNA-binding proteins [15, 16]. In particular, the expression patterns of circRNAs are often tissue- and stage-specific [17], and highly conserved across the human brain and other species [18]. With the fast development of bioinformatics techniques and deep sequencing, a number of circRNAs has been detected in animal follicle development. Tao et al. reports that the host genes of the circRNAs in goat pre-ovulatory ovarian follicles are involved in the ovarian steroidogenesis pathway and the p53 signaling pathway [19], *chi-circ_0008219* modulates follicle growth by sponging three miRNAs to achieve. *circRNA_103827* and *circRNA_104816* detected in human GCs could be a potential indicator of a compromised follicular microenvironment [20]. However, data associated with the function of circRNAs in chicken is still scarce, especially with respect to follicle development. This led us to systematically study the expression of circRNAs in chicken follicle GCs.

In the present study, we performed RNA-seq for circRNAs during chicken follicle development to explore the structure and expression profile of GCs.

## Methods

### Granulosa cells obtained for circular RNA-seq

Sixteen generations of Jinghai Yellow Chicken were reared on Jiangsu Jinghai Poultry Industry Group Co., Ltd. (Nantong City, Jiangsu Province, China). Hens were caged individually while being transferred to the laying house, with a 16 L:8D light regime and ad libitum access to water and a restricted diet. Body weight was measured, and based on the pedigree record, three half-sib hens with an average body weight at 27 weeks of age were humanely sacrificed using 60–70% carbon dioxide. Only hens with a soft eggshell egg in the oviduct were considered in our study. Follicles including SYF (5–8 mm in diameter), hierarchal follicles from smallest to largest including those from F6 to F1 (smallest follicles were 9–12 mm in diameter, largest follicles were ~ 40 mm in diameter), large white follicles (LWF), stroma ovary (O) and postovulatory (Po) follicle, uterus (Ut) and oviduct (Ov) were collected immediately. Detailed methods for collecting follicle GCs and theca cells can be found in a previous paper [21]. We removed the yolk from follicle walls carefully and separated GCs and theca cells, and rinsed them with PBS. The GCs were frozen in liquid nitrogen and used for RNA-seq analysis and experimental validation.

### RNA-seq preparation

Total RNA was isolated by TRIzol reagent (Invitrogen, USA). A series of methods and experimental instruments including 1% agarose gels, Qubit® RNA Assay Kit in Qubit® 2.0 Fluorometer (Life Technologies, USA), the RNA Nano 6000 Assay Kit of the Bioanalyzer 2100 system (Agilent Technologies, USA) and NanoPhotometer® spectrophotometer (IMPLEN, USA) were used to assess the contamination and degradation, concentration, integrity and purity of total RNA.

A total of 5 μg RNA from each sample was used to construct the circRNA library. First, ribosomal RNA was removed using an Epicentre Ribozero™ rRNA Removal Kit (Epicentre, USA). Second, the linear RNA was digested with 3 U of RNase R (Epicentre) per μg of RNA. The sequencing libraries were generated by NEBNext® Ultra™ Directional RNA Library Prep Kit for Illumina® (NEB, USA) following manufacturer’s recommendations. Briefly, first strand cDNA was synthesized using a random hexamer primer and M-MuLV Reverse Transcriptase (RNase H). Second strand cDNA synthesis was subsequently performed using DNA Polymerase I and RNase H. After adenylation of the 3′ ends of DNA fragments, NEBNext Adaptor with hairpin loop structures were ligated to prepare for hybridization. After PCR reaction for fragmentation, and adenylation, products were purified (AMPure XP system) and the library quality was assessed on an Agilent Bioanalyzer 2100 system. Before library preparations were sequenced on the Illumina Hiseq 4000 platform in Novogene (Beijing, China), the cluster generation using TruSeq PE Cluster Kit v3-cBot-HS (Illumina) was performed. Then, 150 bp paired-end reads were generated.

### The circRNA identification and differential expression analysis

Before circRNA identification, quality control was carried out by calculating Q20, Q30, and GC content. Subsequently, paired-end clean reads were aligned to the reference genome using Bowtie [22]. Then, the circRNA were identified by find_circ [23] and CIRI2 [24]. The Circos figure was drawn using Circos software [25].

The raw counts were first normalized using TPM. circRNAs exhibiting fold changes ≥2 with *P-adjust* ≤ 0.05 were classified as circRNAs with significant differential expression.

### Bioinformatics functions analysis

Gene ontology (GO) annotation and Kyoto Encyclopedia of Genes and Genomes (KEGG) pathway analysis were implemented using clusterProfiler package [26] in R software for the host genes of DE circRNA and predicted target genes. MicroRNA target sites in exons of circRNA loci were identified using miRanda (http://www.microrna.org/microrna/home.do) with a score of 140 or higher and free energy − 10 or lower. Target genes of miRNAs were predicted by TargetScan [27] and miRDB [28]. The circRNA-miRNA network was constructed according to the prediction of miRNA binding sites. Cytoscape software was used to construct circRNA-miRNA networks [29].

### Validation of circRNAs by sanger sequencing and qRT-PCR

The circRNAs were validated with convergent and divergent primers according to a previous study [18]. Details of divergent and convergent primers are in Additional file 1: Table S1. PCR products of divergent and convergent primers for cDNA and genomic DNA were analyzed by agarose gel electrophoresis. Back-splicing sites of circRNAs were confirmed by Sanger sequencing at Sango Biotech Co. Ltd. (Shanghai, China).

The expression of PCR products from divergent primers for each GC were validated using qRT-PCR. The qRT-PCR program was implemented using ABI7500 (Life Technologies, USA), with the SYBR Green (TaKaRa, cat. #RR820A) method in a final volume of 20 μL. Each assay was employed in triplicate using the following cycling conditions: 95 °C/30 s and 40 cycles of 95 °C/5 s and 60 °C/34 s. The ^△△^Ct method was used to compare gene expression, with β-actin as a reference gene.

In addition, RNA samples from hierarchal follicles from the F2 to F5 GCs and theca cells, LWF, O, Po, Ut, and Ov were analyzed by qRT-PCR. The expression of circRNAs at different ovary tissues was calculated via the 2^- ΔΔ^ Ct method.

## Results

### Overview of circRNAs

**Fig. 1:**
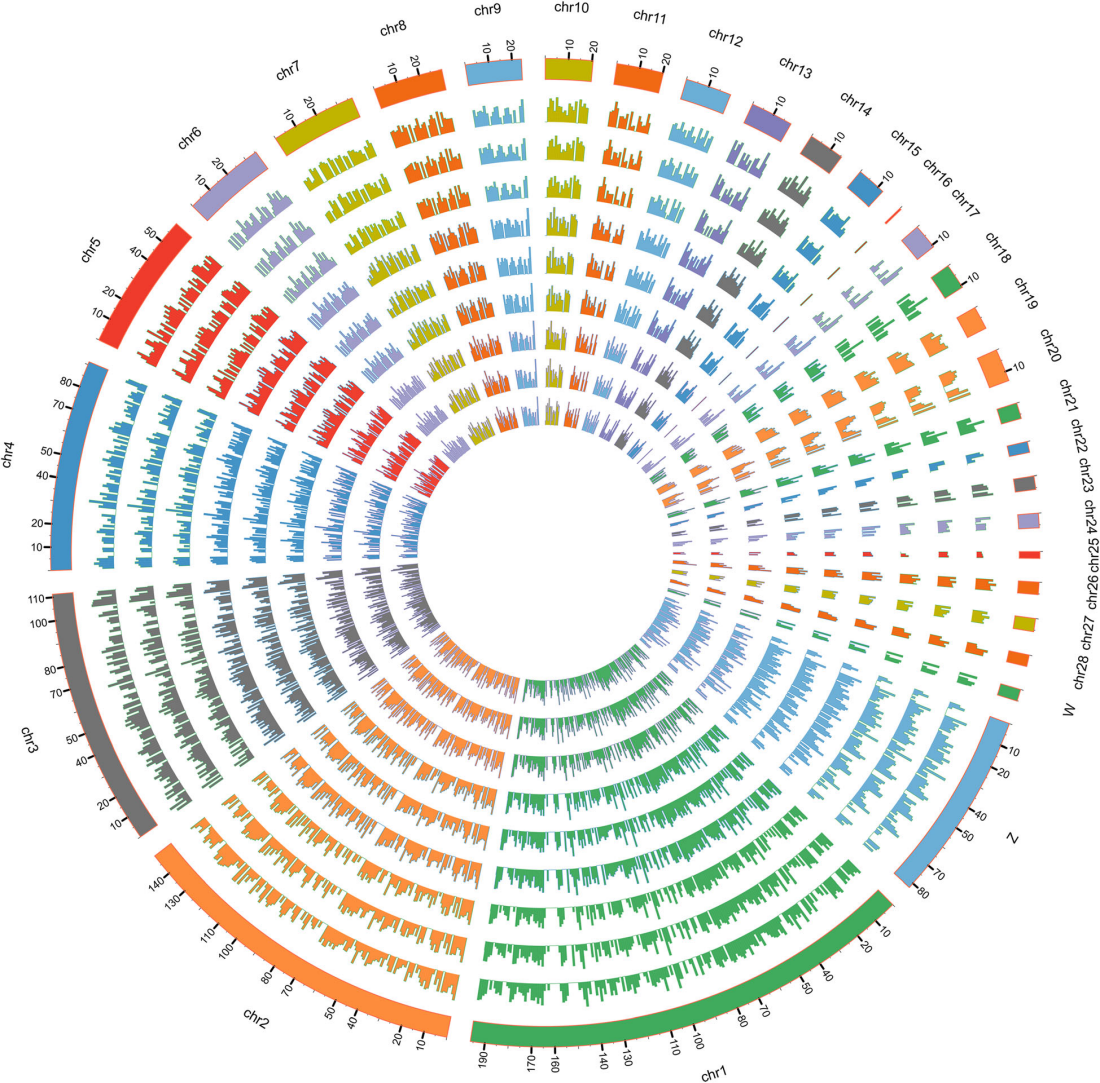
Overview of sequencing data of circRNA by Circos plots. Note: Plot inside are chromosomes from 1 to 28 and sex chromosome converged across granulosa cells (GCs) from SYF (G1, G2, G3), F6 (G1, G2, G3) to F1 (G1, G2, G3), SYF: small yellow follicle; F6: the smallest hierarchal follicles; F1: the largest hierarchal follicle; G: granulosa cell

A total of 864 million raw reads were obtained from RNA-seq data Clean data obtained by removing adapter and low quality sequences were mapped to the chicken reference genome (*Gallus-gallus-5.0/galGal5*). Table 1 summarizes the total number of reads generated from nine samples, and each sample yielded more than 72 million raw reads data. The average GC content was 60.34% (Table 1). Circos figure for circRNAs in GCs is displayed in Fig. 1, which showed that the numbers of circRNAs in GCs were distributed almost on all chromosome, with the largest on chromosome 1 (chr1) and the smallest on the chr16.

**Table 1:** Summary of sequencing results in chicken follicle granulosa cells at three stages

Sample name	Raw reads	Clean reads	Clean bases(G)	Error rate (%)	Q20(%)	Q30(%)	GC content (%)
SYF_G1	131,299,528	126,740,456	19.02	0.02	97.79	94.08	63.83
SYF_G2	82,915,238	80,310,590	12.04	0.02	97.72	94	60.01
SYF_G3	98,659,966	93,718,020	14.06	0.02	98.5	95.84	64.42
F6_G1	99,351,574	96,134,560	14.42	0.03	96.7	91.88	56.73
F6_G2	116,147,306	112,324,754	16.84	0.03	96.56	91.59	56.51
F6_G3	78,903,074	77,034,298	11.56	0.02	97.19	92.84	58.72
F1_G1	86,269,822	84,474,248	12.68	0.03	95.97	90.25	60.02
F1_G2	81,267,114	78,272,912	11.74	0.02	97.38	93.12	60.74
F1_G3	89,875,644	86,894,440	13.04	0.02	97.81	94.09	62.05
Total	864,689,266	835,904,278					60.34

Note: SYF: small yellow follicle, F6: smallest hierarchal follicle, F1: largest hierarchal follicle; G: granulosa cell;

**Fig. 2:**
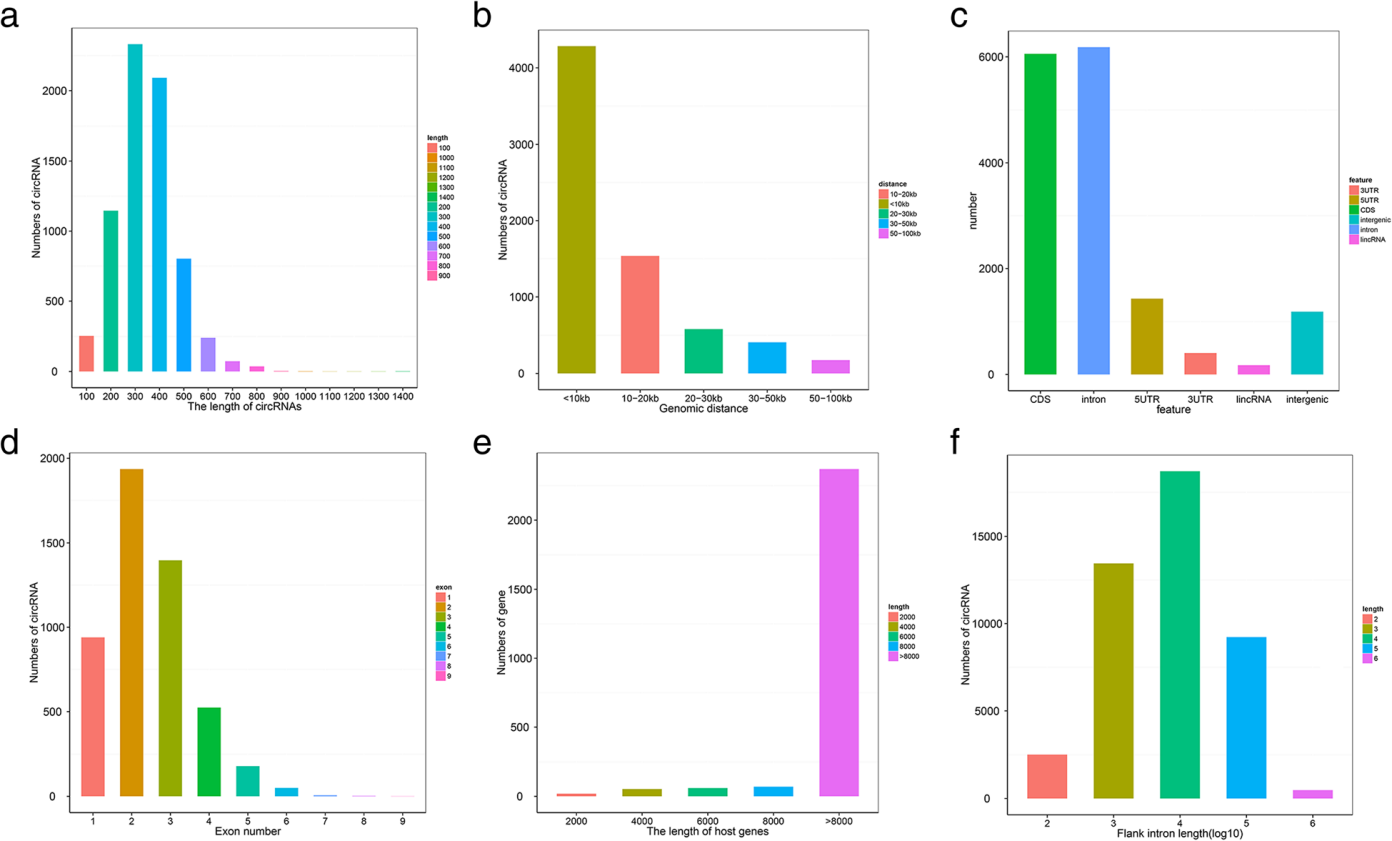
Profiling of circular RNAs in chicken follicle granulosa cells. Note: (**a**), splice length of circRNAs; (**b**), genomic distance between circRNAs; (**c**), source of circRNAs; (**d**), exon numbers; (**e**), length of host genes; (**f**), flank intron length distributed in granulosa cells

After ribosomal-depleted RNA analysis, a total of 11,642 circRNAs were detected by find_circ and CIRI2 (Additional file 1: Table S2). The length of most circRNAs was about 200–300 nt (Fig. 2a) with genomic distances of ~ 1000–3000 bp (Fig. 2b). We grouped circRNAs based on their genomic region including intron, CDS, 5′UTR, intergenic, 3′UTR, and lincRNA. After this step was performed, circRNAs were mainly produced from intron and CDS regions (Fig. 2c). Multiple circRNAs were produced by one gene and the majority of circRNAs have two exons, followed by three exons and one exon (Fig. 2d). Among these CDS sourced circRNAs, about 2371 host gene lengths were more than 8000 nt (Fig. 2e). Approximately, 9165 and 9149 flank introns (42.17%) of circRNAs were above 10^4^~10^5^nt, respectively (Fig. 2f, Additional file 1: Table S3).

### Differentially expressed analysis

**Fig. 3:**
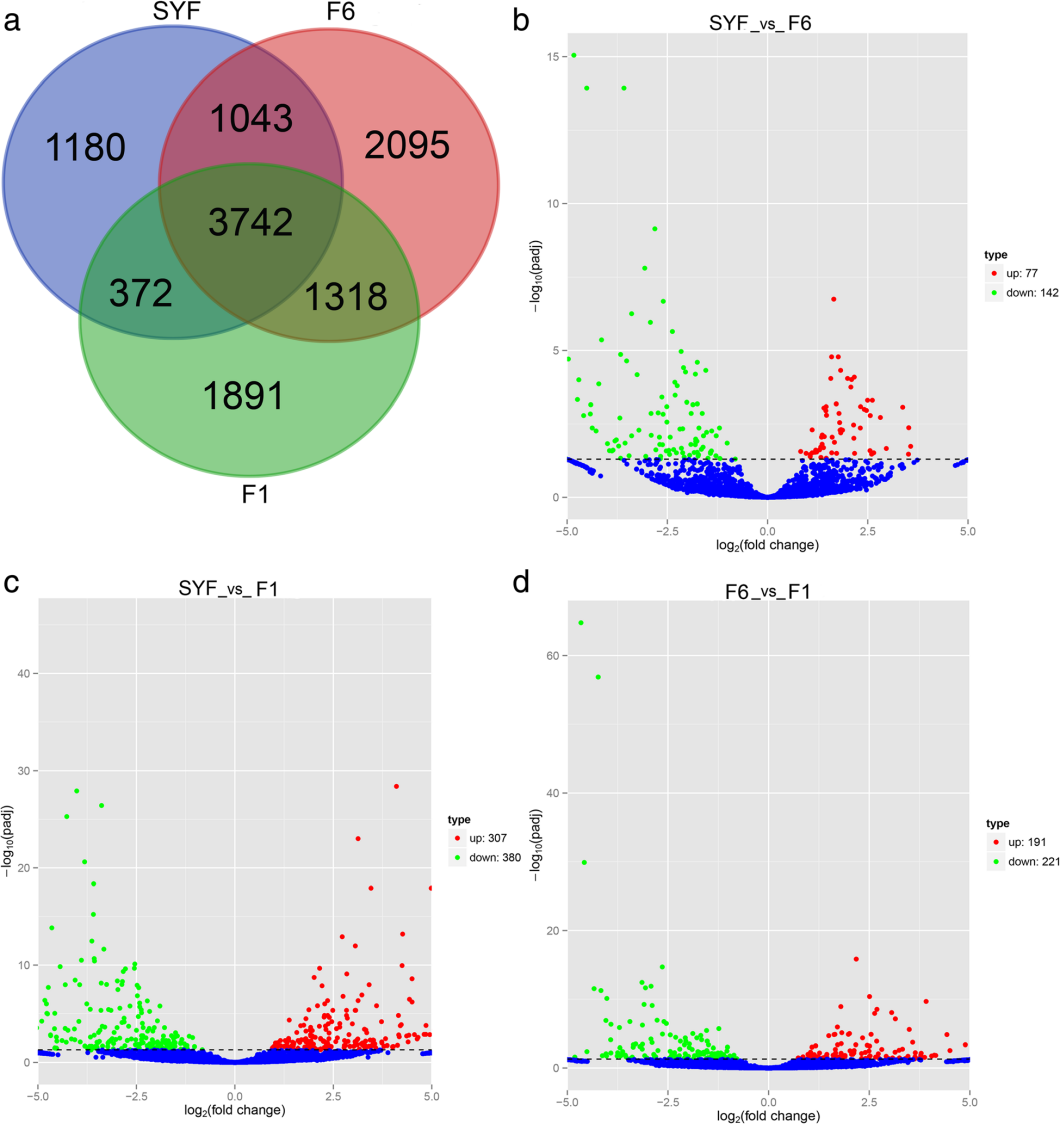
Features of differential expression circRNAs. Note: (**a**), Venn analysis of differentially expressed circRNAs in three stages. **b**, volcano map of differentially expressed circRNAs (DEcircs) between SYF and F6 granulosa cells. **c**, volcano map of DEcircs between SYF and F1 granulosa cells. **d**, volcano map of DEcircs between F6 and F1 granulosa cells. SYF: small yellow follicle; F6: the smallest hierarchal follicles; F1: the largest hierarchal follicle; G: granulosa cell

From the nine GC samples, a total of 6337 circRNAs were in SYF, 8189 in F6, 7323 in F1, and 3742 prevailed in all three follicle GCs (Fig. 3a, Additional file 1: Table S4). Volcano plots of circRNAs expressed differentially in GCs are displayed in Fig. 3b, c, d. In a comparison of SYF and F6 GC libraries, 219 circRNAs were significantly differentially expressed, including 77 upregulated and 142 downregulated (Fig. 3b), while 687 circRNAs were significantly differentially expressed between the SYF and F1 GC libraries (Fig. 3c). In the hierarchal follicle GC libraries, a total of 412 circRNAs with 191 upregulated and 221 downregulated in F6 GCs were significantly differentially expressed (Fig. 3d).

**Fig. 4:**
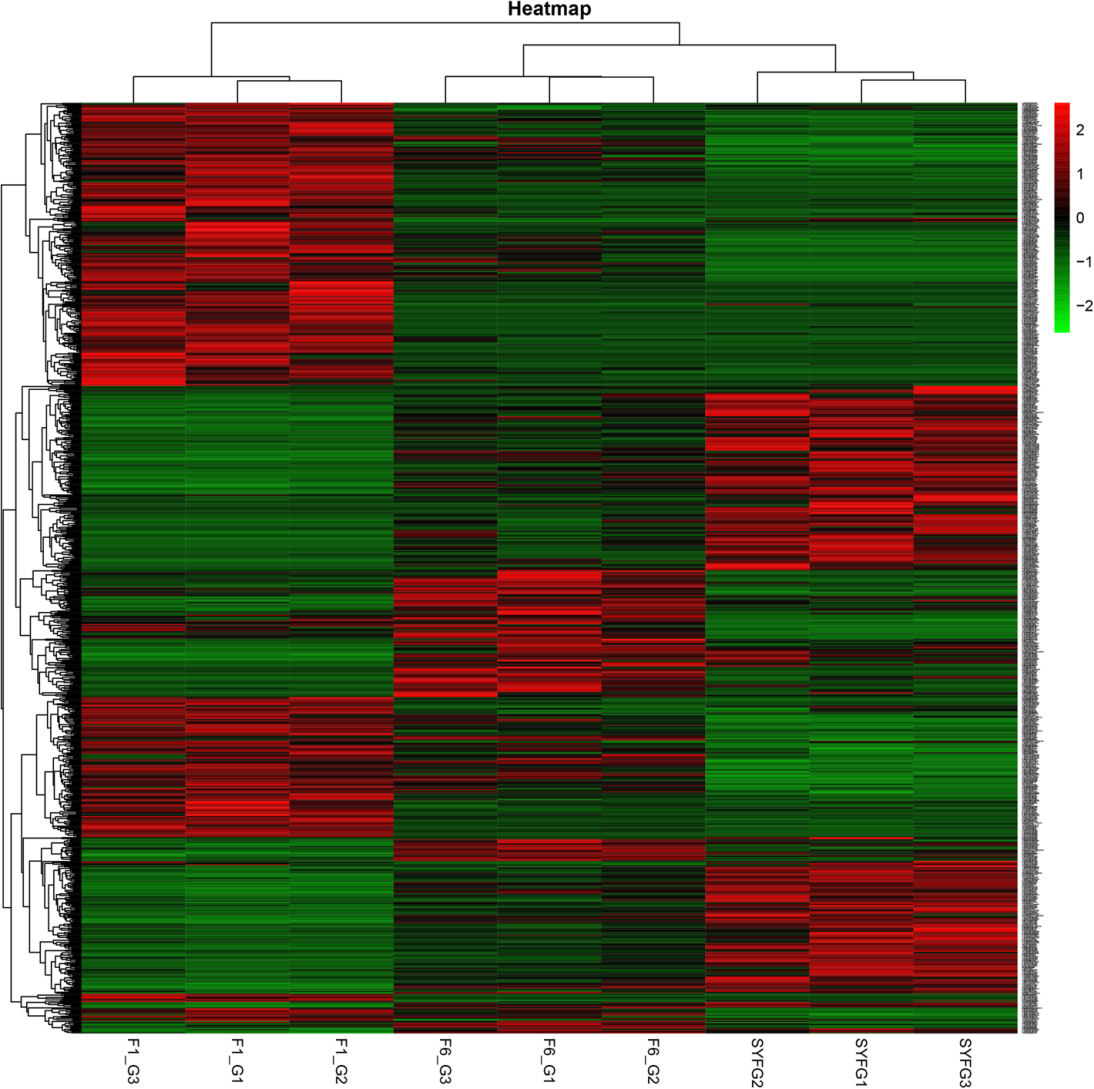
Heat map representing the relative expression levels of all differentially expressed circRNAs in the three stages (Fold change > 2, *P* < 0.05). Note: SYF: small yellow follicle; F6: the smallest hierarchal follicles; F1: the largest hierarchal follicle; G: granulosa cell.

A heatmap of all differentially expressed circRNAs based on transcript per million (TPM) values is shown in Fig. 4. Samples at the same stages are clustered together, the F6_GCs and SYF_GCs are clustered together. The circRNAs changed their expression levels according to the heatmap. In particular, the expression between F1_GCs and SYF_GCs showed the opposite tendency.

### Functional analyses of host genes of differentially expressed circRNAs

**Fig. 5:**
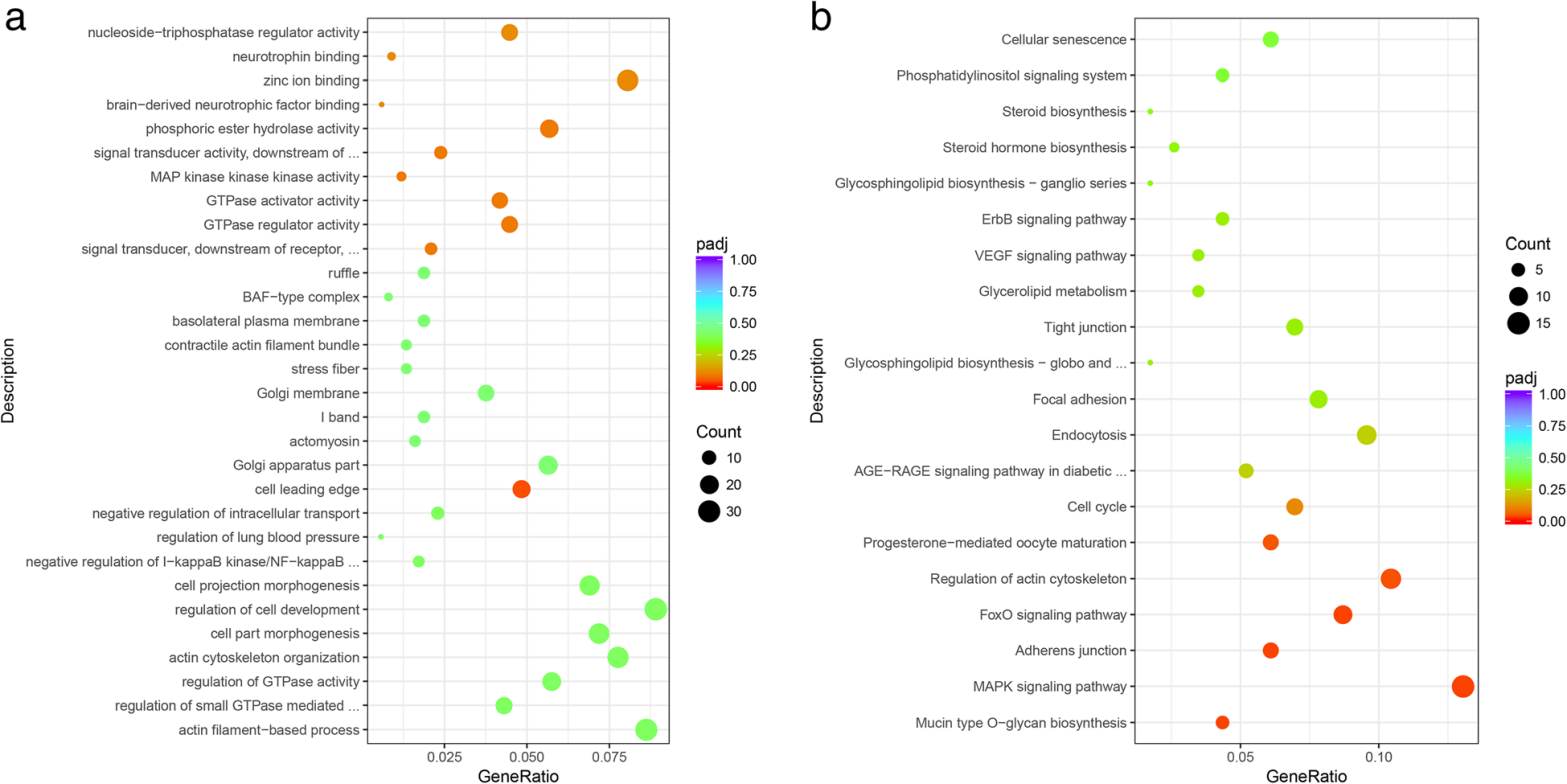
GO and KEGG analysis for the host genes of all differentially expressed circRNAs. Note: (**a**), The top 10 GO enrichment term in BP, CC, and MF for the host genes of all differentially expressed circRNAs; (**b**), the top 20 KEGG enrichment term for the host genes of all differentially expressed circRNAs

The terms from GO of all differentially expressed circRNAs (DEcirc) host genes included GTPase related process and cell development (Additional file 1: Table S5, Fig. 5). The KEGG pathway enrichment of all DEcirc host genes displayed several significant pathways (*P*-adj < 0.05), including the Mucin type O-glycan biosynthesis (4.35%), MAPK signaling pathway (13.04%), Adherens junction (6.09%), FoxO signaling pathway (8.70%), regulation of actin cytoskeleton (10.43%), and progesterone-mediated oocyte maturation (6.09%) (Additional file 1: Table S6).

**Fig. 6:**
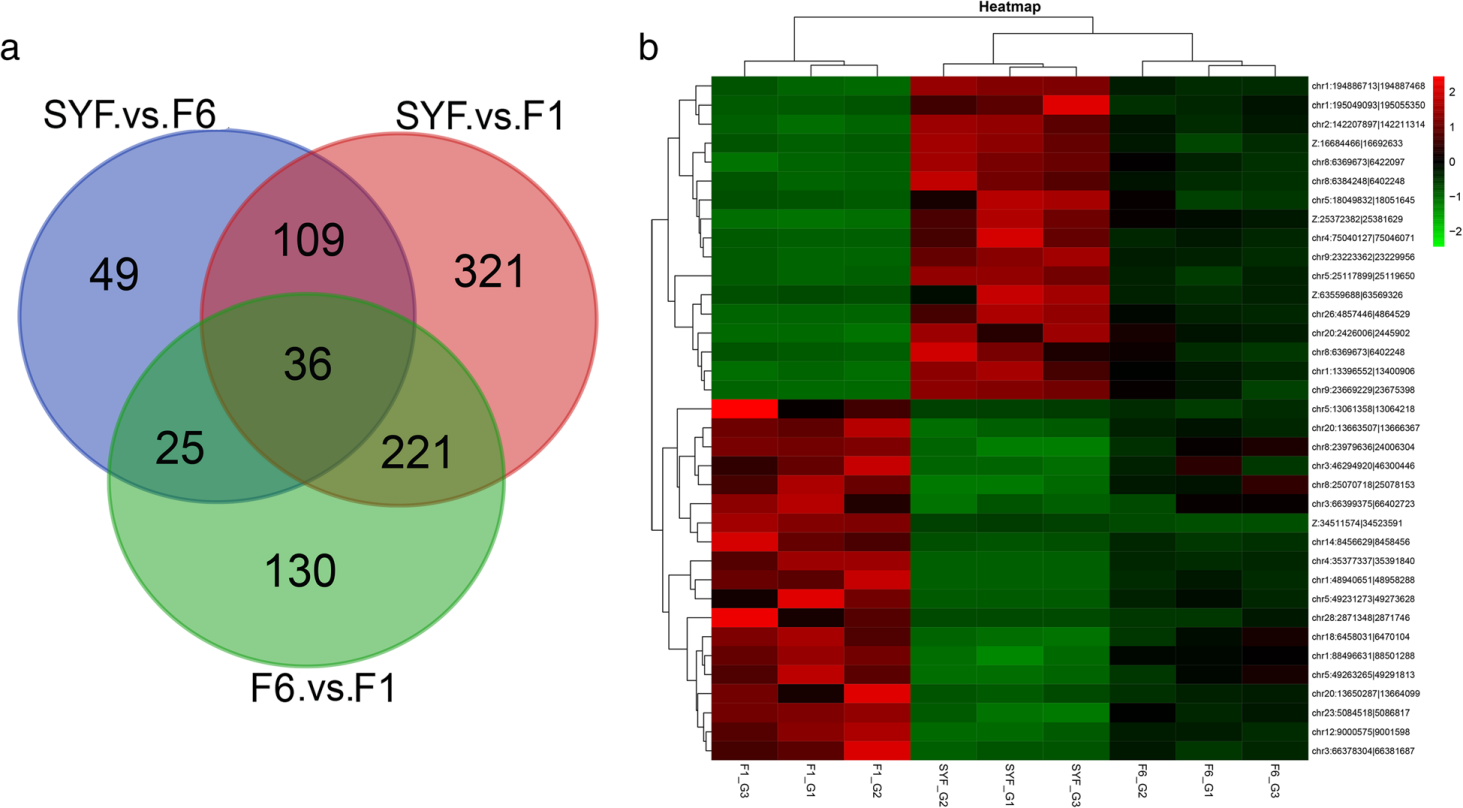
Differentially expressed circRNAs in three different stages of granulosa cells. Note: a, Venn diagram of differentially expressed circRNAs (*n* = 891, fold change > 2, *q-value < 0.05*); b, Heat map of pervasive circRNAs shows expression value at three stages. SYF: small yellow follicle; F6: the smallest hierarchal follicles; F1: the largest hierarchal follicle; G: granulosa cells.

The DE circRNAs between three stages according to Venn analysis are shown in Fig. 6. Notably, 36 pervasive circRNAs existed in three types of GC (Fig. 6a). We clustered the 36 circRNAs based on the relative expression value (Fig. 6b), and the deciphered heat map showed that the expression pattern between SYF and F1 GCs displayed the opposite trend.

### Validation circRNAs and expression pattern

**Fig. 7:**
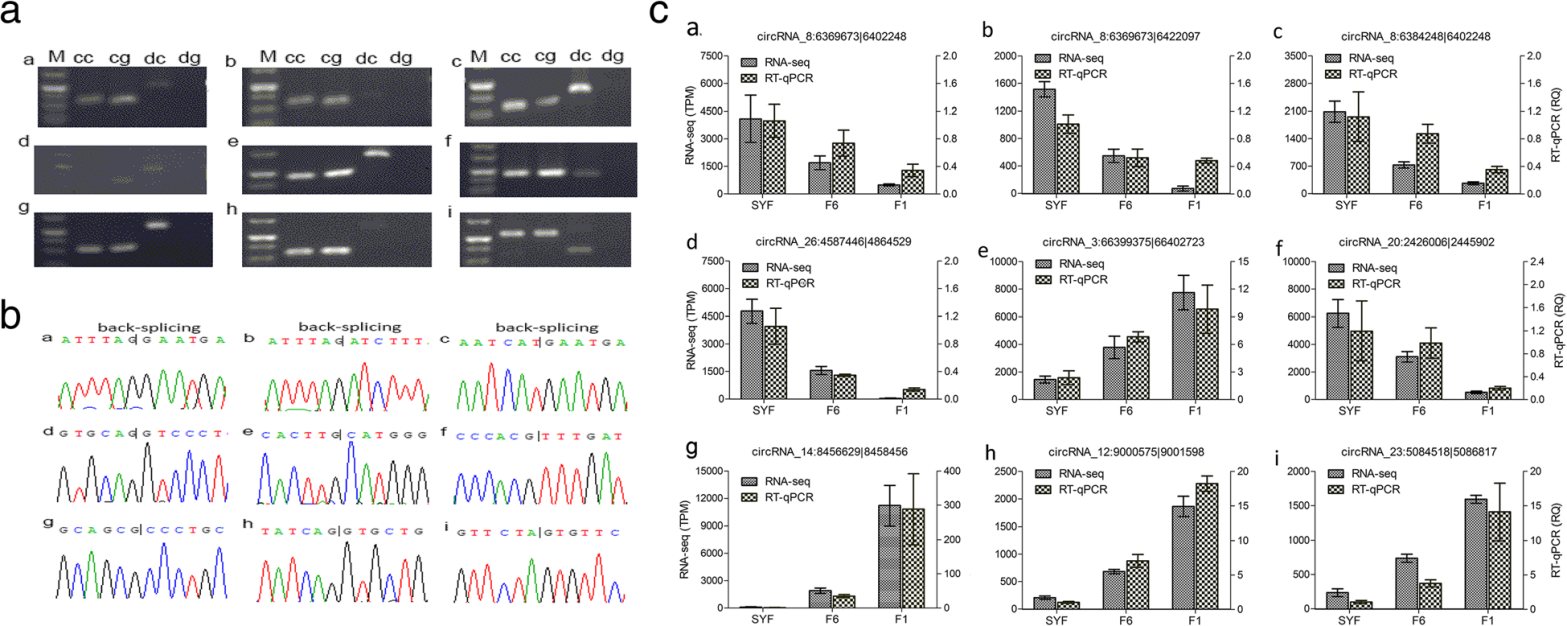
Validation of circRNAs by experimental and sequencing. Note: (**a**), agarose gel electrophoresis test for divergent primers and convergent primers amplify circRNAs; The brightest DNA marker is 200 bp. cc: convergent primers for cDNA; cg: convergent primers for genomic DNA; dc: divergent primers for cDNA; dg: divergent primers for genomic DNA. **b**, Sanger sequencing confirmed the back-splicing junction of circRNAs; (**c**), RT-qPCR validation of nine differentially expressed circRNAs in three types of granulosa cell. TPM: Transcripts Per Million; RQ: relative expression; Graph number of a, b, c, d, e, f, g, h, i represent circRNAs: circRNA_8: 6369673|6,402,248, circRNA_8: 6369673|6,422,097, circRNA_8: 6384248|6,402,248, circRNA_26: 4587446|4,664,529, circRNA_3: 66399375|66,402,723, circRNA_20: 2426006|2,445,902, circRNA_14: 84666291|8,458,456, circRNA_12: 9000575|9,001,598, circRNA_23: 5084518|5,086,817, respectively. SYF: small yellow follicle; F6: the smallest hierarchal follicles; F1: the largest hierarchal follicle; G: granulosa cell.

We randomly confirmed back-site junctions of all chosen circRNAs by agarose gel electrophoresis, Sanger sequencing, and quantitative real-time PCR (qRT-PCR) (Fig. 7). Divergent and convergent primers were designed for each circRNAs, and both genomic DNA (gDNA) and complementary DNA (cDNA) were used as templates for PCR.

Divergent primers from each circRNA amplified the expected fragments by cDNA as a template, while there were no PCR products for the gDNA template, which suggested the presence of back-site junctions (Fig. 7a), which were validated by Sanger sequencing (Fig. 7b). The qRT-PCR validation for the nine circRNAs was consistent with the trends obtained from circRNA sequencing data (Fig. 7c). Notably, three circRNAs, chr8:6369673|6,402,248 (*circRalGPS2_1*), chr8:6369673|6,422,097 (*circRalGPS2_2*), and chr8:6384248|6,402,248 (*circRalGPS2_3*), were from the same gene, *RalGPS2*, and showed pervasive differential expression in three types of GCs.

**Fig. 8:**
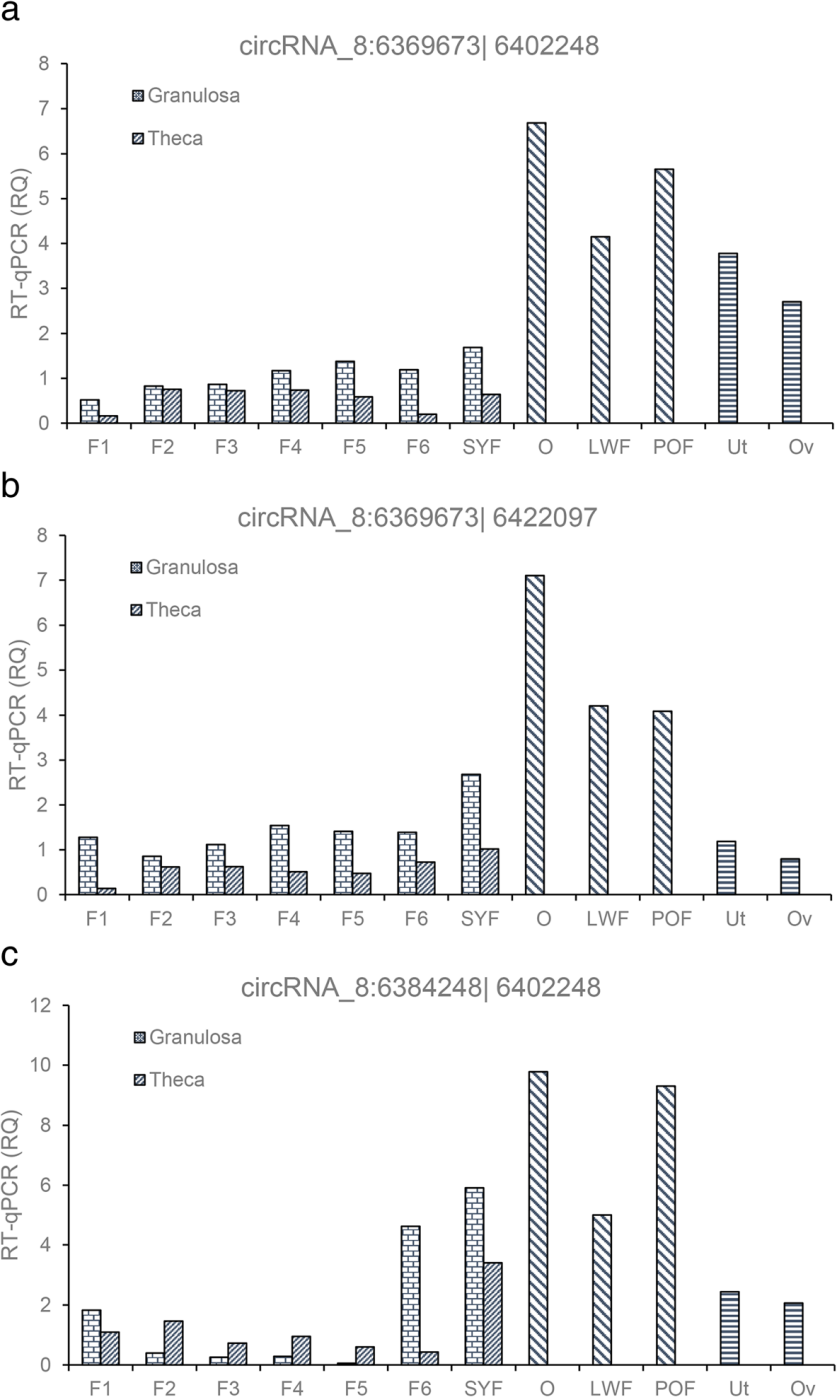
Expression pattern of *circRLAGPS2* during ovarian development Note: F1, F2, F3, F4, F5, F6 represent hierarchal follicles from larger to small; SYF, small yellow follicle; O, ovary; LWF, large white follicle, POF, post-ovulation follicle; Ut, uterus; Ov, oviduct.

Moreover, the expression pattern of *circRalGPS2* in different follicle cells were detected by qRT-PCR, and results are shown in Fig. 8. These three types of circRNAs were all detected in all reproduction tissues. Interestingly, the expression level in the ovary stroma was the highest. The expression level was lower in the hierarchal follicle, GCs, or theca cells compared to the prehierarchal follicle and reproduction tact.

### Target miRNA or gene prediction, networks, and pathways

**Fig. 9:**
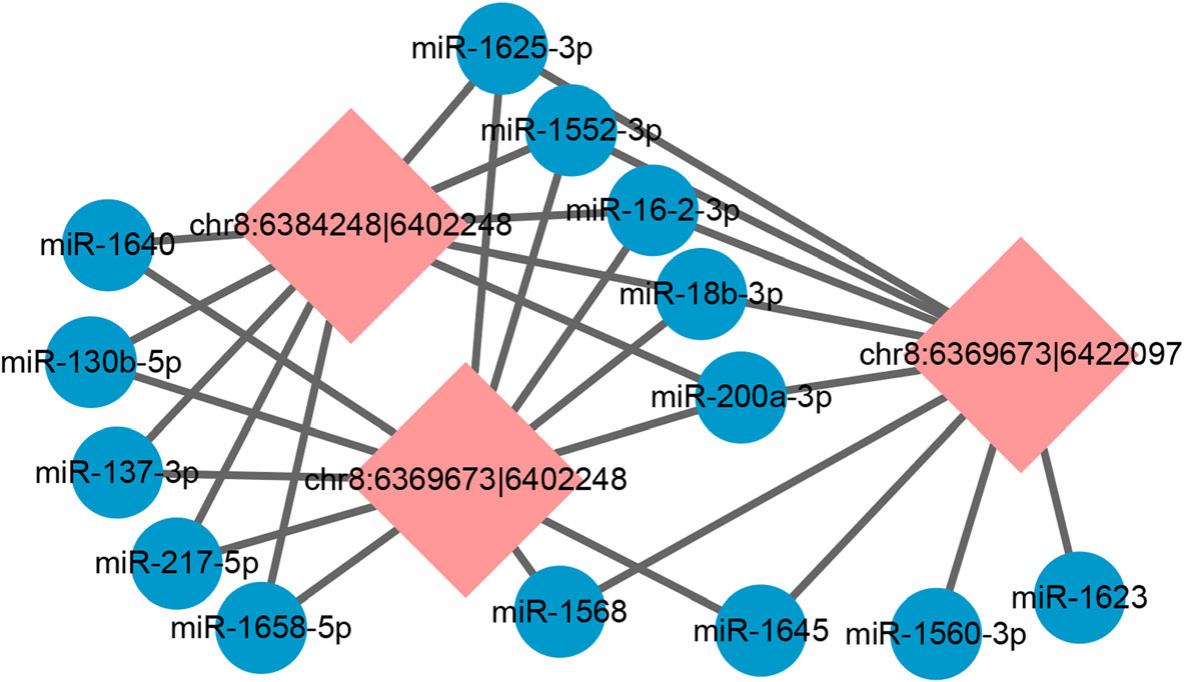
Predicted biomathematical circRNA–miRNA network for the same soured circRNAs. Note: Pink square represents circRNAs, blue circle represents miRNAs.

As demonstrated by a previous study regarding circRNA main functions as a microRNA sponge, we predicted the 36 circRNAs by miRanda based on its sequence. The top five predicted microRNAs are displayed in Table 2. It was observed that five miRNAs (miR-1625-3p, miR-1552-3p, miR-16-2-3p, miR-18b-3p, and miR-200a-3p) were common among the three types of circRNAs sourced from the same gene *RalGPS2* (Fig. 9). A number of overlap genes (538) were predicted by TargetScan and miRDB for these five miRNAs (Additional file 1: Table S6). GO and KEGG analysis was performed for the putative overlap target genes to identify the potential pathways (Additional file 1: Table S8 and S9).

**Table 2:** Thirty-six pervasive DE circRNAs with miRNA binding sites

circRNA_id	Source type	Gene name	Length (nt)	TPM			Predict target miRNA (top 5)
				SYF	F6	F1	
chr1:13396552|13,400,906	exon	*ORC5*	302	5045.66	2279.65	693.55	miR-1620, miR-1620, miR-1596-5p, miR-1596-5p, miR-20b-3p
chr1:194886713|194,887,468	exon	*DGAT2*	296	1039.00	290.59	61.03	miR-301a-3p, miR-9-5p, miR-17-5p, miR-106-5p, miR-1641
chr1:195049093|195,055,350	exon	*GDPD5*	204	845.72	222.16	35.13	miR-17-5p, miR-20a-5p, miR-106-5p,miR-454-3p,miR-215-5p
chr1:48940651|48,958,288	intergenic	*–*	363	0.00	105.17	427.20	miR-1658-3p, miR-1655-5p,miR-1c,miR-15b-5p,miR-15a
chr1:88496631|88,501,288	intergenic	*–*	90	118.63	599.96	1195.79	miR-1631, miR-125b-5p,miR-1611,miR-489-3p,miR-1647
chr12:9000575|9,001,598	exon	*NCKIPSD*	313	203.69	681.61	1861.68	miR-22-5p, miR-1611,miR-183,miR-30c-1-3p,miR-1553-3p
chr14:8456629|8,458,456	exon	*CLEC19A*	390	83.63	1883.10	11,214.67	miR-1550-5p, miR-1594,miR-1635,miR-365-3p,miR-1564-3p
chr18:6458031|6,470,104	intergenic	*–*	417	62.49	641.68	1522.87	miR-1650, miR-1628, miR-20b-3p,miR-219a,miR-1617
chr2:142207897|142,211,314	exon	*ST3GAL1*	375	648.83	227.19	37.71	miR-20a-5p,miR-20b-5p,miR-1649-5p,miR-1553-3p,miR-17-5p
chr20:13650287|13,664,099	intergenic	*–*	277	79.77	404.72	1613.65	miR-217-5p,miR-20a-5p,miR-20b-5p,miR-9-5p,miR-1612
chr20:13663507|13,666,367	intergenic	*–*	413	319.73	1283.93	3535.73	miR-217-5p,miR-142-5p,miR-1646,miR-1612,miR-18a-3p
chr20:2426006|2,445,902	exon	*TOX2*	310	6239.09	3101.73	511.50	miR-1464,miR-1556,miR-1648-3p,miR-140-3p,miR-1614-3p
chr23:5084518|5,086,817	exon	*PTP4A2*	781	233.95	736.32	1591.60	miR-1b-5p,miR-1a-2-5p,miR-1a-1-5p,miR-1565,miR-1649-5p
chr26:4857446|4,864,529	exon	*FOXP4*	287	4771.82	1550.52	47.14	miR-1595-3p,miR-29b-2-5p,miR-1653,miR-460b-5p,miR-29b-1-5p
chr28:2871348|2,871,746	exon	*R3HDM4*	272	0.00	231.30	1373.32	miR-30c-1-3p,miR-1589,miR-181b-5p,miR-181a-5p,miR-1647
chr3:46294920|46,300,446	exon	*UTRN*	328	497.23	2583.92	5557.77	miR-1651-3p,miR-1453,miR-29b-1-5p,miR-1615,miR-138-1-3p
chr3:66378304|66,381,687	exon	*SLC16A10*	481	177.92	620.33	2035.17	miR-1562-5p,miR-215-3p,miR-1625-3p,miR-365-3p,miR-146b-3p
chr3:66399375|66,402,723	exon	*SLC16A10*	786	1447.98	3783.32	7746.36	miR-1585,miR-128-3p,miR-1574-3p,miR-455-3p,miR-124b
chr4:35377337|35,391,840	exon	*FAM13A*	194	0.00	168.06	687.86	miR-1632-5p,miR-29a-5p,miR-1b-3p,miR-1464,miR-100-3p
chr4:75040127|75,046,071	intergenic	*–*	363	2898.18	799.22	28.93	miR-1615,miR-1556,miR-456-3p,miR-142-5p,miR-1609
chr5:13061358|13,064,218	exon	*–*	447	62.81	320.24	4328.23	miR-148a-3p,miR-30a-3p,miR-30e-5p,miR-27b-3p,miR-33-5p
chr5:18049832|18,051,645	exon	*–*	206	2060.95	548.79	95.29	miR-205a,miR-205b,miR-1650,miR-456-3p,miR-19b-5p
chr5:25117899|25,119,650	intergenic	*–*	393	15,111.58	4700.60	1564.80	miR-302a,miR-146a-3p,miR-34a-5p,miR-34c-5p,miR-449a
chr5:49231273|49,273,628	intergenic	*–*	170	0.00	95.43	339.35	miR-1655-5p,miR-1616,miR-30e-3p,miR-1610,miR-1632-5p
chr5:49263265|49,291,813	intergenic	*–*	446	47.11	532.12	1259.60	miR-130a-5p,miR-1570,miR-204,miR-211,miR-1588
chr8:23979636|24,006,304	exon	*FAF1*	606	562.20	1731.65	3037.35	miR-183,miR-1552-3p,miR-26a-5p,miR-1649-5p,miR-302b-5p
chr8:25070718|25,078,153	exon	*–*	198	344.83	2395.12	4397.23	miR-757,miR-15b-5p,miR-15a,miR-16-5p,miR-1630
chr8:6369673|6,402,248	exon	*RALGPS2*	506	4434.04	1688.23	484.23	miR-16-2-3p,miR-137-3p,miR-1645,miR-1640,miR-217-5p
chr8:6369673|6,422,097	exon	*RALGPS2*	484	1513.50	546.63	103.36	miR-16-2-3p,miR-1645,miR-1625-3p,miR-18b-3p,miR-1560-3p
chr8:6384248|6,402,248	exon	*RALGPS2*	371	2084.74	728.96	262.57	miR-16-2-3p,miR-137-3p,miR-1640,miR-217-5p,miR-1658-5p
chr9:23223362|23,229,956	intergenic	*–*	811	2474.39	703.18	56.05	miR-130b-5p,miR-130b-5p,miR-106-3p,miR-106-3p,miR-17-3p
chr9:23669229|23,675,398	exon	*MED12L*	647	5842.60	2133.05	307.58	miR-15b-5p,miR-15a,miR-16-5p,miR-20b-5p,miR-7
Z:16684466|16,692,633	exon	*CDC20B*	540	1527.75	326.33	47.14	miR-1640,miR-1605,miR-215-3p,miR-1617,miR-1560-5p
Z:25372382|25,381,629	intron	*–*	292	1969.73	914.92	54.04	miR-9-5p,miR-1649-5p,miR-1571,miR-1592,miR-499-5p
Z:34511574|34,523,591	exon	*PIP5K1B*	359	1252.09	494.63	12,054.15	miR-125b-3p,miR-1564-3p,miR-1620,miR-181b-2-3p,miR-1574-5p
Z:63559688|63,569,326	exon	*SSBP2*	284	4447.64	1002.17	111.02	miR-301a-3p,miR-1550-3p,miR-130b-3p,miR-551-5p,miR-200a-5p

Note: SYF: small yellow follicle, F6: smallest hierarchal follicle, F1: largest hierarchal follicle

The results showed that a total of 145 predicted target genes were annotated to 92 pathways (Additional file 1: Table S9). Among these, pathways involving tight junctions and oocyte meiosis were significantly enriched (*P-adj < 0.05*). Other pathways involving in *GnRH* signaling, *Wnt* signaling, MAPK signaling pathway, progesterone-mediated oocyte maturation, *TGF-beta signaling pathway*, and *FoxO* signaling were related to animal reproduction. The enriched genes related to reproduction pathways inclu*ded MAP3K5, ADCY3*, *PLCB1*, *CAMK2G*, *PIK3R1*, and another 37genes (Additional file 1: Table S8).

## Discussion

The field of chicken reproductive function biology is still in its infancy and many questions remain. If hens can produce longer layer periods, they could be taken full advantage of and economic benefits would improve. Investigations into the GCs of follicles could provide information underlying follicle development and make further improvements in egg production. Moreover, other applications could include a model to study the molecular events that are the most common fate of ovarian follicles [30]. Folliculogenesis in birds is a very complex process, which includes recruitment of the primitive follicle, development or atresia of small follicles and 6–8 mm follicle selection, hierarchal follicle rapid growth, and ovulation. Knowledge of developmental changes during the folliculogenesis process in chicken can be incorporated into egg production and has important ramifications for understanding ovarian function with chicken as a model organism [30, 31]. The circRNA and polyA-RNAs have rich and extremely important functions on a variety of biological processes [23]. Though many studies have reported that circRNAs were widespread in mammals [32] and plants [14], little is known about circRNAs in chickens, especially in the folliculogenesis process.

In the current study, a strategy of second generation sequencing for circRNAs in chicken follicle GCs was performed. The circRNAs were widely distributed in chromosomes 1–28, 30, 32–33 Z, W, and two linkage groups (Fig. 1**,** Additional file 1: Table S1), which suggested that circRNAs had complexity and functional diversity. The circRNAs distributed on chr1 were the largest, while those on chr16 were the smallest, which was in line with the chromosome length. GGA1 is the longest chromosome in the entire genome of the chicken and chr16 is the shortest [33]. We predicted a total of 11,642 circRNAs in three types of follicle GC, a number which is less than that detected in the muscle of embryonic chicken (13,377) [34] and more than that found in the liver of ALVJ-resistant and susceptible chicken (1800) [35], which suggested that the expression of circRNAs were tissue-specific and assisted in the hypothesis that the circularization is a tightly regulated event in different tissues [32]. In our study, the spliced length of the majority of circRNAs was 200~300 nt, which was longer than those obtained in a previous study from pre-implantation embryos (124–227 nt) [36]. In total, 94.83% of spliced circRNAs were less than 500 nt, which was higher than in other studies of circRNA expression in rat liver (45.56%) [37]. The difference of length in different animals may be due to a species difference in the function performed by circRNAs. With respect to the source of circRNAs in GCs, it was shown that circRNAs mainly came from introns, which was unlike in previous research where most circRNAs came from coding exons [34]. Other aspects about the production rate or exon number in circRNAs were similar with previous studies, for instance, the production rate of circRNAs is determined by intronic sequence [38] and a single gene can produce multiple circRNAs [39]. When all the above features on circRNA are combined, it was concluded that not only was there a species difference in circRNA expression, which was tissue-specific, but common features were also shared.

With respect to differential expression in circRNAs, Fig. 3 shows that GC circRNAs exhibited stage-specific expression, which is in line with reports in human and mouse [32]. The most abundant expression was in F6_GCs with 2095 stage-specific expression circRNAs, followed by F1_GCs. The fluctuation of circRNA abundance in GCs may be related to their specific roles during the development of follicles.

We drew a heat map for all differential expression circRNAs according to its TPM (Fig. 4), which indicated that these circRNAs have a dynamic expression pattern during GC growth. Notably, the expression level between F6_GCs and SYF_GCs showed an opposite tendency, and these two GCs have different cell morphologies during follicle development; small follicles contain several layers of GCs whereas mature follicles have a single layer of GCs [30]. It was concluded that circRNAs may exert different functions on different cell morphology of the same tissue.

Moreover, GO and KEGG pathway analyses for these differentially expressed circRNAs were performed. Most GO terms were enriched in GTPase related processes and cell developmental processes. GTPases of the Ras superfamily are omnipresent signaling proteins that play important roles in a wide range of vital cellular processes [40], including ovarian follicle development [41, 42]. A study showed that the activation of GTPases is critical for the regulation of cell junctions and cell-cell adhesion [43] and it was shown to be responsible for the accumulation of cell junction proteins between oocytes and GCs [41]. Tight junctions between oocytes and GCs are dynamically regulated in a developmental stage-specific manner [44]. Some GTPase proteins also regulate GC proliferation, differentiation and follicle selection in chicken [42]. Taken together, these observations and our results indicate that those circRNAs are involved in GCs progression and communications between oocyte and GCs. Moreover, several other pathways related to reproduction are also significantly enriched including pathways involved in *M*APK signaling, FoxO signaling, and progesterone-mediated oocyte maturation. Similar results were observed in noncoding RNAs including long non-coding RNA and microRNA enrichment analysis in previous studies [45, 46], which suggest that these circRNAs may act as competing endogenous RNAs (ceRNAs) to modulate gene transcription. Findings obtained from our study and previous reports suggest that follicle development is a complex process that includes numerous events, and circRNAs distributed on three types of follicle GCs are mainly involved in tight junctions to participate in follicle growth, reproduction events, and other biological processes.

As demonstrated in Fig. 6, a total of 36 differentially expressed circRNAs showed pervasive expression patterns in three types of GCs. Interestingly, expression levels presented an increased or decreased trend with the development of follicle GCs. Previous work has reported that the abundance of circRNAs were negatively correlated with proliferation in cancer tissue compared to normal tissue [47] and circularization of circRNAs can compete with canonical pre-mRNA splicing [38]. The correlation of circRNA expression level and the quality of embryo numbers were reported to be negative in Quan et al. [48]. It is tempting to speculate that these upregulated circRNAs may inhibit GC proliferation and downregulated circRNAs may affect follicle number by participating in the regulation of follicle recruitment.

We observed that three circRNA isoforms (*circRalGPS2_1*, *circRalGPS2_2*, and *circRalGPS2_3*) from the same host gene *RalGPS2* (known as a hot spot gene [49]) widely distributed on three GCs and displayed similar differential expression trend, which is unlike previous reports where they displayed dissimilar expression profiles [49]. Presumably, these circRNAs are generated from one particular exon that is common to *RalGPS2*. It remains to be validated whether circRNAs from the same back-splicing can perform a similar expression level to modulate the transcription process. In addition, previous research showed that *RalGPS2* may play a role in cytoskeleton reorganization [50] and is responsible for survival and the cell cycle in lung cancer cells [51]. To date, few data have been published regarding the role of *RalGPS2* in follicle GCs, whether it is GC-type-specific as with mRNA. Interestingly, *RalGPS2* was enriched in many GTPase processes in our study (Additional file 1: Table S4). We examined the expression changes of *circRalGPS2s* during follicle growth. The results showed that the expression of all *circRalGPS2s* were predominant in the ovary stroma and a decreased level was found in follicle growth, suggesting that circularization of circRNAs may not accompany GCs development. It has been proven that circRNAs such as *circRNA_103827* and *circRNA_104816* expression level is negatively related to embryonic development [20]. With the combined analysis of the abovementioned reports and current results, it is plausible to hypothesize that the lower the level of circRNAs, the more abundant are the display of biological activities, and circRNA from different host genes may exert different functions in different tissues, organs, or stages.

**Fig. 10:**
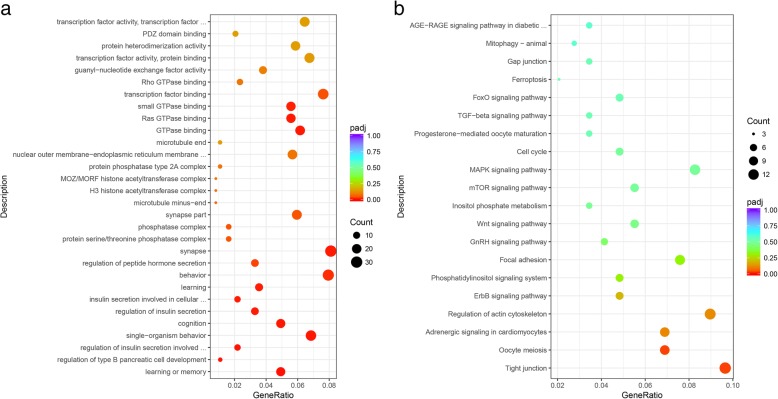
GO and KEGG analysis of *circRalGPS2-miRNA* target genes. Note: (**a**), Gene ontology of target genes. **b**, KEGG pathways of target genes.

The main mechanism of circRNAs may act as a miRNA sponge [16] to modulate post-transcriptional regulation [23]. The higher level of circRNAs, the stronger is the efficiency of the sponge [16]. The circRNA-miRNA network (Fig. 9) displayed that circRNAs generated from the same gene may regulate common target miRNA that bound to the same genes to perform its function. A study about human GCs showed that the predicted target genes of *circRNA_103827* and *circRNA_104816* are mainly enriched in glucose metabolism, the mitotic cell cycle, and ovarian steroidogenesis [20]; these pathways play a key role in follicle development. GO and KEGG analysis for the predicted target genes of *circRalGPS2* showed that the target genes were enriched in the regulation of signaling, secretion by the cell, and cell motility (Fig. 10a), suggesting that circRNAs may be potentially responsible for these functional areas. KEGG bioinformatics analysis results indicated that *circRalGPS2s* were significantly involved in pathways of tight junctions and oocyte meiosis. Tight junctions were also enriched in DE circRNA annotations, which further explain the role of tight junctions as a key pathway in follicle development. Other pathways related to reproduction were also enriched, such as *GnRH* signaling, the *Wnt* signaling pathway, *mTOR* signaling, *MAPK* signaling, progesterone-mediated oocyte maturation, and *FoxO* signaling. Genes enriched in many pathways include *ADCY3 (ENSGALG00000016608)*, *PIK3R1 (ENSGALG00000014786)*, *PAK3 (ENSGALG00000008058)*, and *CAMK2G (ENSGALG00000005088)*, which have been reported to play important roles during reproduction processes. For example, knock-out of *ADCY3* leads to the resumption of gamete mitosis [52]. *PIK3R1* is involved in the *PI3K/AKT* pathway that is responsible for chicken GC proliferation [53]. *CAMK2A* (the paralog gene of *CAMK2G*), and *PAK3* are located in a putative QTL region that is required for increasing broiler follicle number [54]. Although the function of these genes in chicken granulosa development is unclear, previous reports indicate that they play important roles in regulating reproduction. Furthermore, on the basis of the ceRNA hypothesis [55], *circRalGPS2* can compete with miRNA to modulate mRNA expression that regulates downstream target genes. Follicle development is a complex process, with the susceptibility of multiple genes or transcriptional factors as well as the interactions between them. Thus, an assessment of the function of circRNAs as a miRNA sponge to modulate gene expression during follicle development needs tentative overexpression and knockdown experimental designs.

## Conclusions

In summary, our study is the first to elucidate spatiotemporal comprehensive circRNA expression profile in follicle GCs of chicken. These results from RNA-seq for circRNAs suggested that circRNA are abundant, with tissue-specific and stage-specific expression in chicken follicle GCs. In particular, we found a hot spot gene *RalGPS2* that produced three isoforms of circRNAs which exhibited a similar expression pattern during follicle development. Furthermore, the function of these circRNAs needs to be investigated by experimental study. However, these findings could be helpful to decipher the regulatory mechanisms underlying follicle growth and engineer practical breeding programs.

## Additional files

Additional file 1: **Table S1.** Detailed information of primers used in this study. **Table S2.** All novel circRNAs detected in granulosa cells. **Table S3.** Flank intron length statistics. **Table S4.** Differentially expressed circRNAs (Fold change > 2, q-value < 0.05) in different granulosa cells. **Table S5.** Gene ontology for host genes of differential expressed circRNAs. **Table S6.** KEGG pathway for host genes of all differentially expressed circRNAs in three granulosa cells stage. **Table S7.** Overlapped prediction genes by TargetScan and miRDB. **Table S8.** Gene ontology of predicted target genes based on circRALGPS2. **Table S9.** KEGG pathway of predicted target genes based on circRALGPS2. (XLSX 1280 kb)

## Abbreviations

BP: Biological process
CC: Cell component
cDNA: Complementary DNA
ceRNA: Competing endogenous RNA
DE: Differential expression
F1: The largest hierarchal follicle of ~ 40 mm in diameter
F6: The smallest hierarchal follicle of 9–12 mm in diameter
GC: Granulosa cell
gDNA: Genomic DNA
GO: Biological process in gene ontology
KEGG: Kyoto Encyclopedia of Genes and Genomes
MF: Molecular function
O: Ovary
Ov: Oviduct
Po: Postovulatory
qRT-PCR: Quantitative real-time PCR
SYF: Small yellow follicle
TPM: Transcripts Per Million
Ut: Uterus
